# Possible diverse contribution of coronary risk factors to left ventricular systolic and diastolic cavity sizes

**DOI:** 10.1038/s41598-021-81341-1

**Published:** 2021-01-15

**Authors:** Kenichiro Suzuki, Yasunori Inoue, Kazuo Ogawa, Tomohisa Nagoshi, Kosuke Minai, Takayuki Ogawa, Makoto Kawai, Michihiro Yoshimura

**Affiliations:** Division of Cardiology, Department of Internal Medicine, The Jikei University School of Medicine, 3-25-8 Nishi-shinbashi, Minato-ku, Tokyo, 105-8461 Japan

**Keywords:** Anatomy, Cardiology, Risk factors

## Abstract

It is generally believed that risk factors damage the coronary arteries, cause myocardial ischemia, and consequently change the shape of the heart. On the other hand, each of the risk factors may also have a negative effect on the heart. However, it is very difficult to examine the effects of each of these risk factors independently. Therefore, it is necessary to select an appropriate statistical method and apply it efficiently. In this study, the effects of coronary risk factors on left ventricular size and cardiac function were investigated using structure equation modeling (SEM), and were shown as Bayesian SEM-based frequency polygons using selected two-dimensional contours. This study showed that each risk factor directly affected the shape of the heart. Because vascular risk and heart failure risk are likely to evolve at the same time, managing risk factors is very important in reducing the heart failure pandemic.

## Introduction

When cardiac overload continues due to prolonged myocardial ischemia and/or myocardial infarction, the heart causes expansion of the left ventricular (LV) cavity sizes, which conforms to the Frank-Starling Law^1^. On the other hand, there are only a few reports examining the direct contribution of conventional risk factors of ischemic heart disease (IHD), such as aging, male sex, obesity, hypertension, smoking, diabetes, dyslipidemia and others^2–4^, on the change in LV cavity sizes.

Based on the knowledge that abnormal metabolic process induced by some risk factors may produce myocardial metabolic inefficiency, we hypothesized that the respective coronary risk factors would have a high potential for directly affecting LV cavity sizes not only through the intermediary of atherosclerotic heart disease induced by the risk factors. On a relevant note, oxidative stress induced by some risk factors may also be another important process for reducing cardiac function and changing cardiac sizes. Not only myocardial damage but also interstitial cell damage, such as interstitial fibrosis, may be indispensable in the argument for this hypothesis.

There are many unresolved points to discuss about the relative size of the impact of coronary risk factors on LV cavity sizes. In particular, if there are some pathological effects of the respective risk factors on LV cavity sizes, it would be important to discuss whether the respective risk factors result in LV enlargement or LV reduction. More specifically, it is important to examine which of the respective risk factors exert an influence on systolic LV cavity size or diastolic LV cavity size. With regard to LV cavity size, the effect of respective risk factors on LV ejection fraction (LVEF) should also be examined.

It is, however, quite difficult to study how respective risk factors influence LV cavity sizes. Set out below are the possible reasons. The risk factors are statistically confounded with each other, and the true effect of each risk factor on LV cavity sizes can hardly be evaluated. Also, since the systolic volume and the diastolic volume are strongly associated with each other as a matter of course, it is difficult to determine which phase of systole or diastole the respective risk factors mainly influence. It is thus ideal that the real effects of respective risk factors should be examined simultaneously in one equation model. That study method would be difficult assignment from a statistical perspective, however.

Herein, we suppose that structure equation modeling (SEM) or covariance structure analysis would fit the current purpose. In many areas, SEM plays an important role in understanding how the relationship among observed variables might be generated by an effect of interaction. Recently, we constructed several ideal models of a path diagram due to the solution of some disease status^5–14^. In this study, we tried to examine the effect of respective risk factors, such as aging, gender difference, body mass index (BMI), hypertension, diabetes, and dyslipidemia, on LV cavity sizes at the timing of systole and diastole in patients with IHD by using SEM. In addition, to reassess the current data from another statistical perspective, Bayesian SEM was also applied.

## Results

### Study patients’ characteristics

Table 1 shows the characteristics of the study patients.

**Table 1:** Clinical characteristics.

Characteristic	Overall (n = 1556)
	Number (%) or mean ± SD or median [interquartile range]
Gender, male	1373 (88.2)
Age (years old)	66.2 ± 10.4
BMI (kg/m^2^)	24.8 ± 3.6
Current + past smoker	1088 (69.9)
Family history of IHD	462 (29.7)
Hb (g/dL)	13.6 ± 1.7
Creatinine (mg/dL)	0.85 ± 0.40
eGFR (mL/min/1.73 m^2^)	71.7 ± 16.2
UA (mg/dL)	5.9 ± 1.3
FBS (mg/dL)	117.9 ± 30.1
HbA1c (%)	6.4 ± 1.0
TG (mg/dL)	128,7 ± 101.9
HDL-C (mg/dL)	50.7 ± 14.1
LDL-C (mg/dL)	96.3 ± 26.6
LDL-C/HDL-C	2.04 ± 0.79
CRP (mg/dL)	0.60 [0.30–0.22]
BNP (pg/mL)	30.9 [15.0–77.4]
**Left ventricular hemodynamic parameters**	
LVEDVI (mL/m^2^)	62.0 ± 17.6
LVESVI (mL/m^2^)	26.2 ± 14.1
LVEF (%)	59.1 ± 10.1
LVEDP (mmHg)	13.3 ± 6.8
SVI (mL/m^2^)	35.8 ± 8.6
**Underlying cardiovascular disease**	
Stable angina pectoris	1556 (100)
Old myocardial infarction	532 (34.2)
Coronary spastic angina	33 (2.1)
Cardiomyopathy	30 (19.3)
Arrhythmia	119 (7.6)
Atrial fibrillation	61 (3.9)
Other than AF	58 (3.7)
Hypertension	1192 (76.6)
Type 2 diabetes mellitus	683 (43.9)
Dyslipidemia	1266 (81.4)
Renal dysfunction	336 (21.6)
**Vessel disease**	
0-VD, n (%)	738 (47.4)
1-VD, n (%)	482 (31.0)
2-VD, n (%)	218 (14.0)
3-VD, n (%)	118 (7.6)
**Medication**	
Antiplatelet agent	1439 (92.5)
Anticoagulant agent	171 (11.0)
ACE inhibitors	395 (25.4)
ARBs	658 (42.3)
Aldosterone blockers	141 (9,1)
Beta blockers	724 (46.5)
Calcium channel blockers	963 (61.9)
Diuretics	258 (16.6)
Statins	1205 (77.4)
Non-statin for dyslipidemia	223 (14.3)
Oral antidiabetic agents	480 (30.8)
Insulin	146 (9.4)
Anti-hyperuricemia	248 (15.9)

*SD* standard deviation, *ACE* angiotensin-converting enzyme, *AF* atrial fibrillation, *ARBs* angiotensin receptor blockers, *BMI* body mass index, *BNP* B-type natriuretic peptide, *CRP* C-reactive protein, *eGFR* estimate glomerular filtration rate, *IHD* ischemic heart disease, *FBS* fasting blood sugar, *Hb* hemoglobin, *UA* uric acid, *HbA1c* hemoglobin A1c, *TG* triglyceride, *HDL-C* high-density lipoprotein cholesterol, *LDL-C* low-density lipoprotein cholesterol, *LVEDP* left ventricular end-diastolic pressure, *LVEF* left ventricular ejection fraction, *LVEDVI* left ventricular end-diastolic volume index, *LVESVI* left ventricular end-systolic volume index, *SVI* stroke volume index.

### Associations between LV cavity volume and coronary risk factors

**Figure 1:**
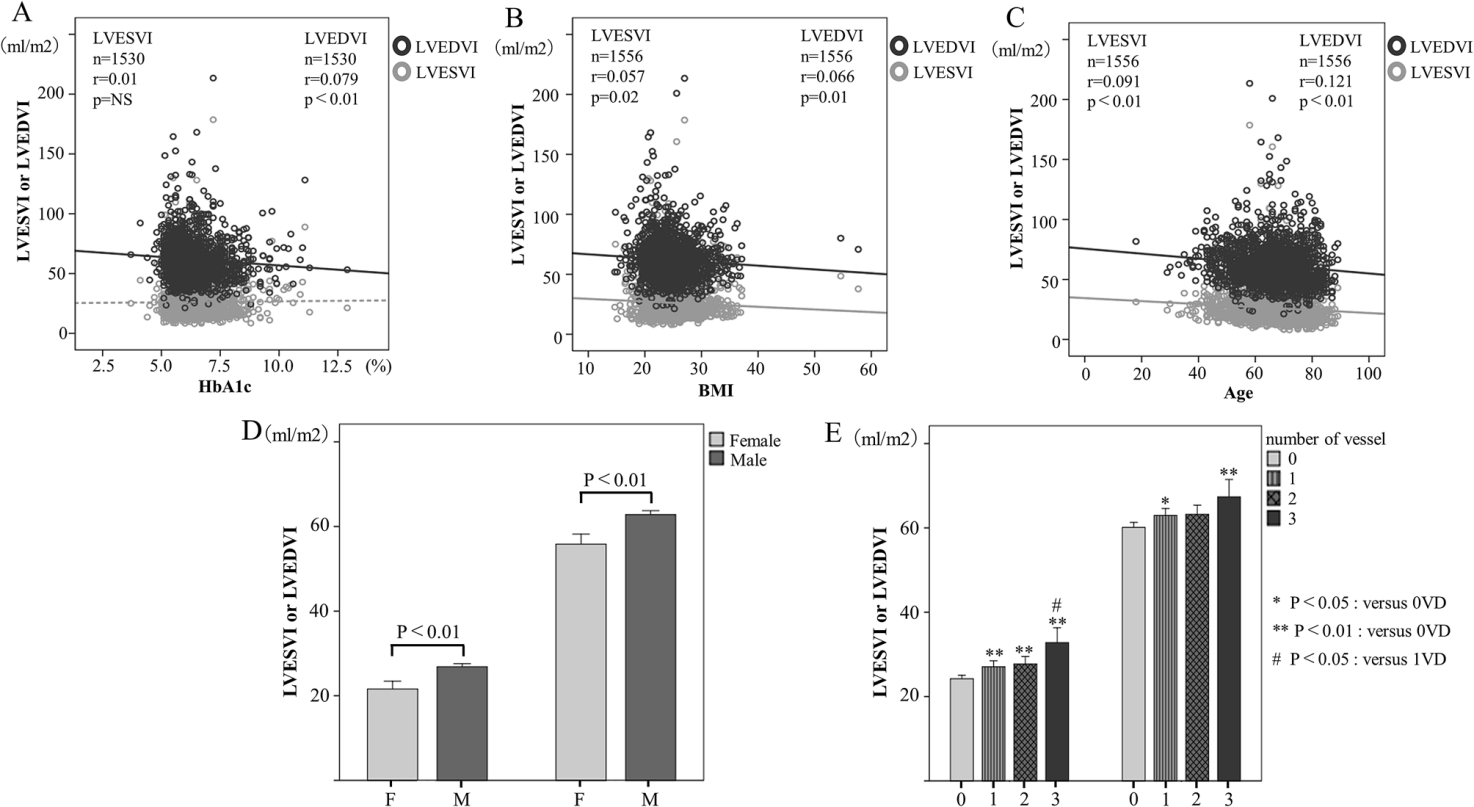
Associations and comparison of LV cavity volume with HbA1c, BMI, Age, gender and number of diseased vessels. (**A**) HbA1c, (**B**) BMI, (**C**) Age, (**D**) Gender, (**E**) Number of diseased vessels. The LV cavity volume and coronary risk factors are represented as scatter plots (**A–C**). The gray plots are LVESVI and the black plots are LVEDVI. The solid gray or black line indicates the regression curve for the logarithmic fitted equation. The bar chart of LV cavity size is shown by gender, or by number of diseased vessels (**D**,**E**). *P < 0.05, **P < 0.01 versus 0 vessel disease at another vessel disease group. ^#^P < 0.05, versus 1 vessel disease at another vessel disease group. *BMI* body mass index, *HbA1c* hemoglobin A1c, *LVESVI* left ventricular end-systolic volume index, *LVEDVI* left ventricular end-diastolic volume index.

Figure 1A–E shows the associations between LV cavity volume [LV end-systolic volume index (LVESVI), LV end-diastolic volume index (LVEDVI)] and coronary risk factors [BMI, age, hemoglobin Alc (HbA1c), gender, number of diseased vessels]. The LVEDVI was significantly negative correlated with HbA1c, BMI and age (P < 0.05, respectively). Whereas the LVESVI was significantly negative correlated with BMI and age (P < 0.05, respectively). Impressively, HbA1c was not correlated with LVESVI. Male had significantly higher LVEDVI and LVESVI values than female (respectively, P < 0.001). The number of diseased vessels were increasing LVEDVI and LVESVI values.

### Concept of the proposed path model A (for affecting LVESVI and LVEDVI)

**Figure 2:**
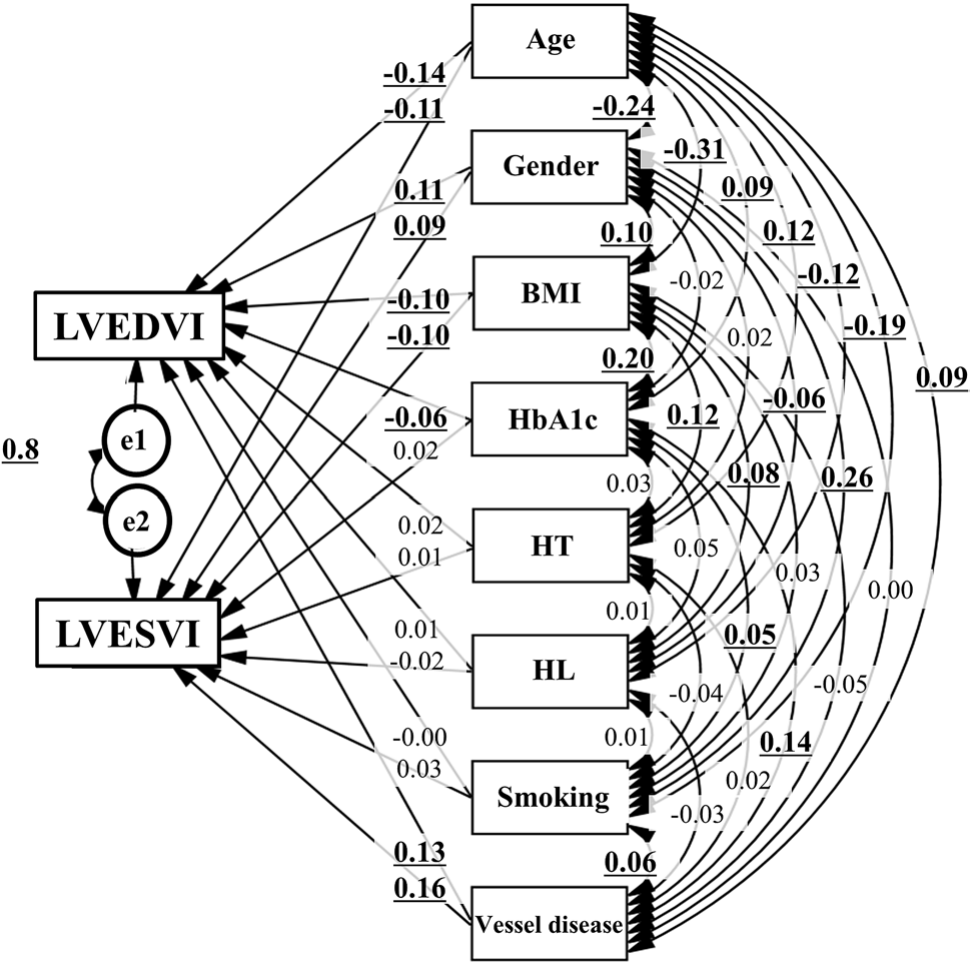
Path model (A): An association of the coronary risk factors with LVESVI and LVEDVI with explanatory drawing of possible cascade from the respective risk factors. This path shows the standardized coefficient of regressing independent variables on the dependent variable of the relevant path. These variables indicate standardized regression coefficients, squared multiple correlations (italic capitals), and correlations among exogenous variables (capitals upper side of the two-way arrowhead curves). *BMI* body mass index, *HT* hypertension, *HbA1c* hemoglobin A1c, *HL* hyperlipidemia, *LVESVI* left ventricular end-systolic volume index, *LVEDVI* left ventricular end-diastolic volume index.

Path model A was proposed, as shown in Fig. 2. Paths between variables were drawn from independent to dependent variables with a directional arrow for the regression model. This was based on whether the respective risk factors themselves causatively induced LVESVI and LVEDVI. To indicate what is critical, the respective risk factors are linked by bidirectional arrows. The same applies to the link between LVESVI and LVEDVI.

### Results of path model A (for affecting LVESVI and LVEDVI)

Table 2 shows the detailed results of the SEM from Fig. 2. Severely stenosed coronary arteries increased LVESVI and LVEDVI (*P* < 0.001, respectively). Aging reduced LVESVI and LVEDVI (*P* < 0.001, respectively). BMI reduced LVESVI and LVEDVI (*P* < 0.001, respectively). Male sex increased LVESVI and LVEDVI (*P* < 0.001, respectively). However, diabetes showed a different trend; hemoglobin Alc reduced only LVEDVI (*P* = 0.019) but did not affect LVESVI (*P* = NS). A history of hyperlipidemia, smoking, and hypertension affected neither LVESVI nor LVEDVI.

**Table 2:** Results of the path model described in Fig. 1

Clinical factor			Estimate	Standard error	Test statistic	P value	Standard regression coefficient		
							Direct effect	Indirect effect	Total effect
LVEDVI	←	Age	− 0.233	0.047	− 4.906	< 0.001	− 0.137	0	− 0.137
(mL/m^2^)	←	Gender	5.728	1.433	3.997	< 0.001	0.105	0	0.105
	←	BMI	− 0.495	0.131	− 3.772	< 0.001	− 0.103	0	− 0.103
	←	HbA1c	− 1.118	0.475	− 2.352	0.019	− 0.061	0	− 0.061
	←	HT	0.758	1.05	0.722	0.47	0.018	0	0.018
	←	HL	0.278	1.13	0.246	0.806	0.006	0	0.006
	←	Smoking	− 0.105	0.65	− 0.162	0.871	− 0.004	0	− 0.004
	←	IHD	2.417	0.469	5.149	< 0.001	0.129	0	0.129
LVESVI	←	Age	− 0.155	0.038	− 4.095	< 0.001	− 0.114	0	− 0.114
(mL/m^2^)	←	Gender	4.136	1.147	3.605	< 0.001	0.094	0	0.094
	←	BMI	− 0.378	0.105	− 3.595	< 0.001	− 0.098	0	− 0.098
	←	HbA1c	0.292	0.381	0.768	0.442	0.02	0	0.02
	←	HT	0.181	0.84	0.216	0.829	0.005	0	0.005
	←	HL	− 0.741	0.905	− 0.819	0.413	− 0.02	0	− 0.02
	←	Smoking	0.495	0.52	0.951	0.342	0.025	0	0.025
	←	IHD	2.466	0.376	6.564	< 0.001	0.164	0	0.164
Age	↔	BMI	− 11.776	1.004	− 11.73	< 0.001			− 0.311
Age	↔	HbA1c	0.905	0.256	3.528	< 0.001			0.09
Age	↔	HT	0.541	0.112	4.823	< 0.001			0.123
Age	↔	HL	− 0.466	0.103	− 4.52	< 0.001			− 0.115
Age	↔	Smoking	− 1.411	0.188	− 7.49	< 0.001			− 0.193
Age	↔	IHD	0.891	0.248	3.588	< 0.001			0.091
Age	↔	Gender	− 0.793	0.087	− 9.117	< 0.001			− 0.238
BMI	↔	HbA1c	0.686	0.091	7.503	< 0.001			0.195
BMI	↔	HT	0.189	0.039	4.792	< 0.001			0.122
BMI	↔	HL	0.112	0.036	3.103	0.002			0.079
BMI	↔	Smoking	0.088	0.065	1.351	0.177			0.034
BMI	↔	IHD	− 0.165	0.087	− 1.896	0.058			− 0.048
BMI	↔	Gender	0.111	0.03	3.718	< 0.001			0.095
HbA1c	↔	HT	0.011	0.01	1.056	0.291			0.027
HbA1c	↔	HL	0.02	0.01	2.055	0.04			0.053
HbA1c	↔	Smoking	0.034	0.017	1.966	0.049			0.05
HbA1c	↔	IHD	0.124	0.023	5.313	< 0.001			0.137
HbA1c	↔	Gender	− 0.007	0.008	− 0.875	0.381			− 0.022
HT	↔	HL	0.001	0.004	0.332	0.74			0.008
HT	↔	Smoking	− 0.013	0.008	− 1.685	0.092			− 0.043
HT	↔	IHD	0.006	0.01	0.632	0.527			0.016
HT	↔	Gender	0.002	0.003	0.593	0.553			0.015
HL	↔	Smoking	0.004	0.007	0.505	0.613			0.013
HL	↔	IHD	− 0.013	0.009	− 1.358	0.174			− 0.034
HL	↔	Gender	− 0.008	0.003	− 2.442	0.015			− 0.062
Smoking	↔	IHD	0.037	0.017	2.203	0.028			0.056
Smoking	↔	Gender	0.06	0.006	10.053	< 0.001			0.264
IHD	↔	Gender	0	0.008	− 0.018	0.986			0
e1	↔	e2	205.051	7.895	25.973	< 0.001			0.875

*BMI* body mass index, *HbA1c* hemoglobin A1c, *HL* hyperlipidemia, *HT* hypertension, *IHD* ischemic heart disease, *LVEDVI* left ventricular end-diastolic volume index, *LVESVI* left ventricular end-systolic volume index.

### Concept of the proposed path model B (for affecting LVEF)

Next, we evaluated the effects of respective risk factors on LVEF. Path model B was proposed for this aim. By the same token, paths between variables were drawn from independent to dependent variables with a directional arrow for the regression model.

### Results of path model B (for affecting LVEF)

**Figure 3:**
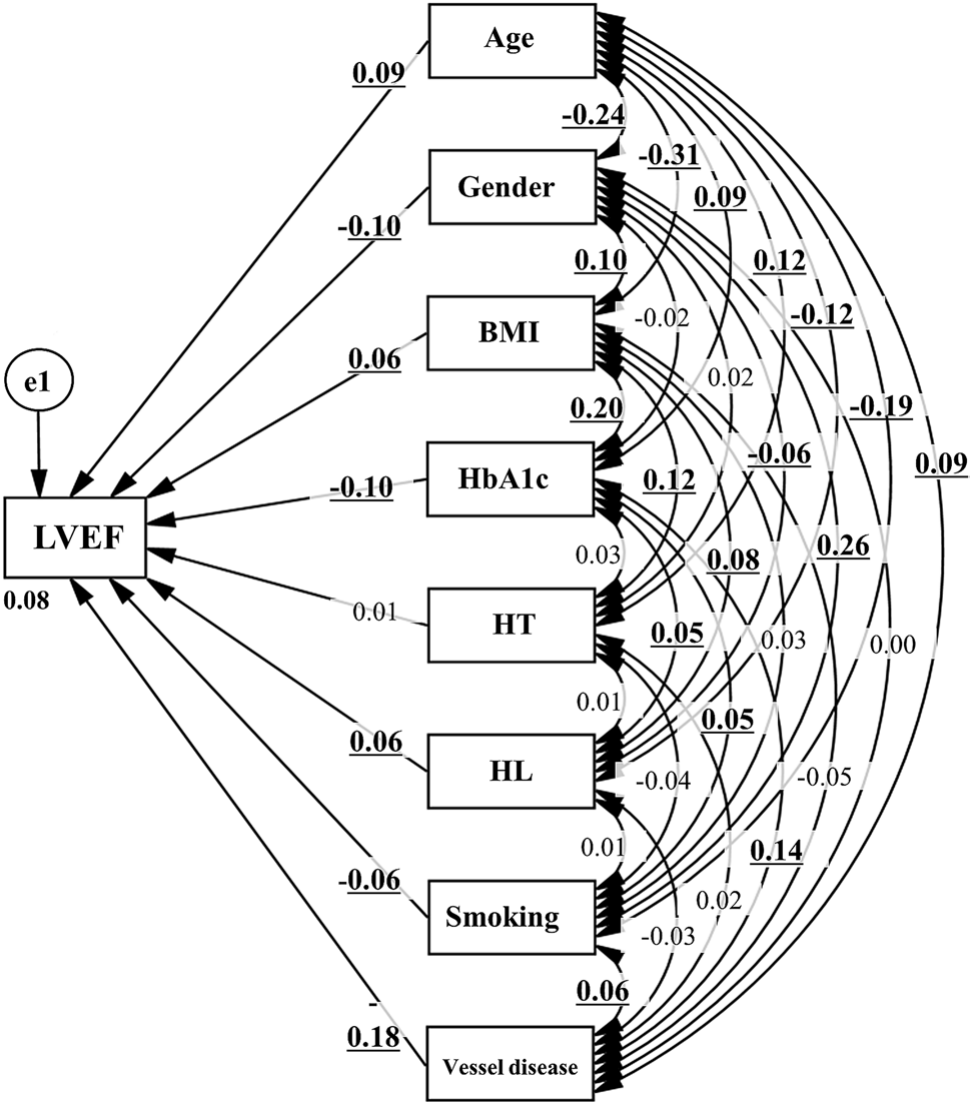
Path model (B): An association of the coronary risk factors with LVEF with explanatory drawing of possible cascade from the respective risk factors. This path shows the standardized coefficient of regressing independent variables on the dependent variable of the relevant path. These variables indicate standardized regression coefficients, squared multiple correlations (italic capitals), and correlations among exogenous variables (capitals upper side of the two-way arrowhead curves). *BMI* body mass index, *HT* hypertension, *HbA1c* hemoglobin A1c, *HL* hyperlipidemia.

Table 3 shows the detailed results of SME from Fig. 3. The high number of stenosed coronary arteries reduced LVEF (*P* < 0.001). Aging and BMI increased LVEF (*P* < 0.001, respectively). Male sex decreased LVEF (*P* < 0.01), or to put it another way, female sex increased LVEF (*P* < 0.01). HbA1c significantly decreased LVEF (*P* < 0.001). We may add that smoking status decreased LVEF (*P* = 0.025) and that a history of hyperlipidemia increased LVEF (*P* = 0.011). A history of hypertension did not affect LVEF in this study.

**Table 3:** Results of the path model (B).

Clinical factor			Estimate	Standard error	Test statistic	*P* value	Standard regression coefficient		
							Direct effect	Indirect effect	Total effect
LVEF	←	Age	0.091	0.027	3.404	< 0.001	0.094	0	0.094
(%)	←	Gender	− 3.109	0.81	− 3.837	< 0.001	− 0.099	0	− 0.099
	←	BMI	0.167	0.074	2.249	0.024	0.06	0	0.06
	←	HbA1c	− 1.073	0.269	− 3.992	< 0.001	− 0.103	0	− 0.103
	←	HT	0.329	0.594	0.555	0.579	0.014	0	0.014
	←	HL	1.666	0.639	2.607	0.009	0.064	0	0.064
	←	Smoking	− 0.908	0.367	− 2.472	0.013	− 0.063	0	− 0.063
	←	IHD	− 1.907	0.265	− 7.185	< 0.001	− 0.178	0	− 0.178
Age	↔	BMI	− 11.776	1.004	− 11.73	< 0.001			− 0.311
Age	↔	HbA1c	0.903	0.256	3.52	< 0.001			0.09
Age	↔	HT	0.541	0.112	4.823	< 0.001			0.123
Age	↔	HL	− 0.466	0.103	− 4.52	< 0.001			− 0.115
Age	↔	Smoking	− 1.411	0.188	− 7.49	< 0.001			− 0.193
Age	↔	IHD	0.891	0.248	3.589	< 0.001			0.091
Age	↔	Gender	− 0.793	0.087	− 9.117	< 0.001			− 0.238
BMI	↔	HbA1c	0.686	0.091	7.5	< 0.001			0.195
BMI	↔	HT	0.189	0.039	4.792	< 0.001			0.122
BMI	↔	HL	0.112	0.036	3.103	0.002			0.079
BMI	↔	Smoking	0.088	0.065	1.351	0.177			0.034
BMI	↔	IHD	− 0.165	0.087	− 1.894	0.058			− 0.048
BMI	↔	Gender	0.111	0.03	3.718	< 0.001			0.095
HbA1c	↔	HT	0.011	0.01	1.062	0.288			0.027
HbA1c	↔	HL	0.02	0.01	2.08	0.038			0.053
HbA1c	↔	Smoking	0.034	0.017	1.972	0.049			0.05
HbA1c	↔	IHD	0.124	0.023	5.307	< 0.001			0.137
HbA1c	↔	Gender	− 0.007	0.008	− 0.882	0.378			− 0.023
HT	↔	HL	0.001	0.004	0.332	0.74			0.008
HT	↔	Smoking	− 0.013	0.008	− 1.684	0.092			− 0.043
HT	↔	IHD	0.006	0.01	0.631	0.528			0.016
HT	↔	Gender	0.002	0.003	0.593	0.553			0.015
HL	↔	Smoking	0.004	0.007	0.505	0.613			0.013
HL	↔	IHD	− 0.013	0.009	− 1.359	0.174			− 0.034
HL	↔	Gender	− 0.008	0.003	− 2.442	0.015			− 0.062
Smoking	↔	IHD	0.037	0.017	2.206	0.027			0.056
Smoking	↔	Gender	0.06	0.006	10.053	< 0.001			0.264
IHD	↔	Gender	0	0.008	− 0.019	0.985			0

*BMI* body mass index, *HbA1c* hemoglobinA1c, *HL* hyperlipidemia, *HT* hypertension, *IHD* ischemic heart disease, *LVEF* left ventricular ejection fraction.

### Results of Bayesian SEM

**Figure 4:**
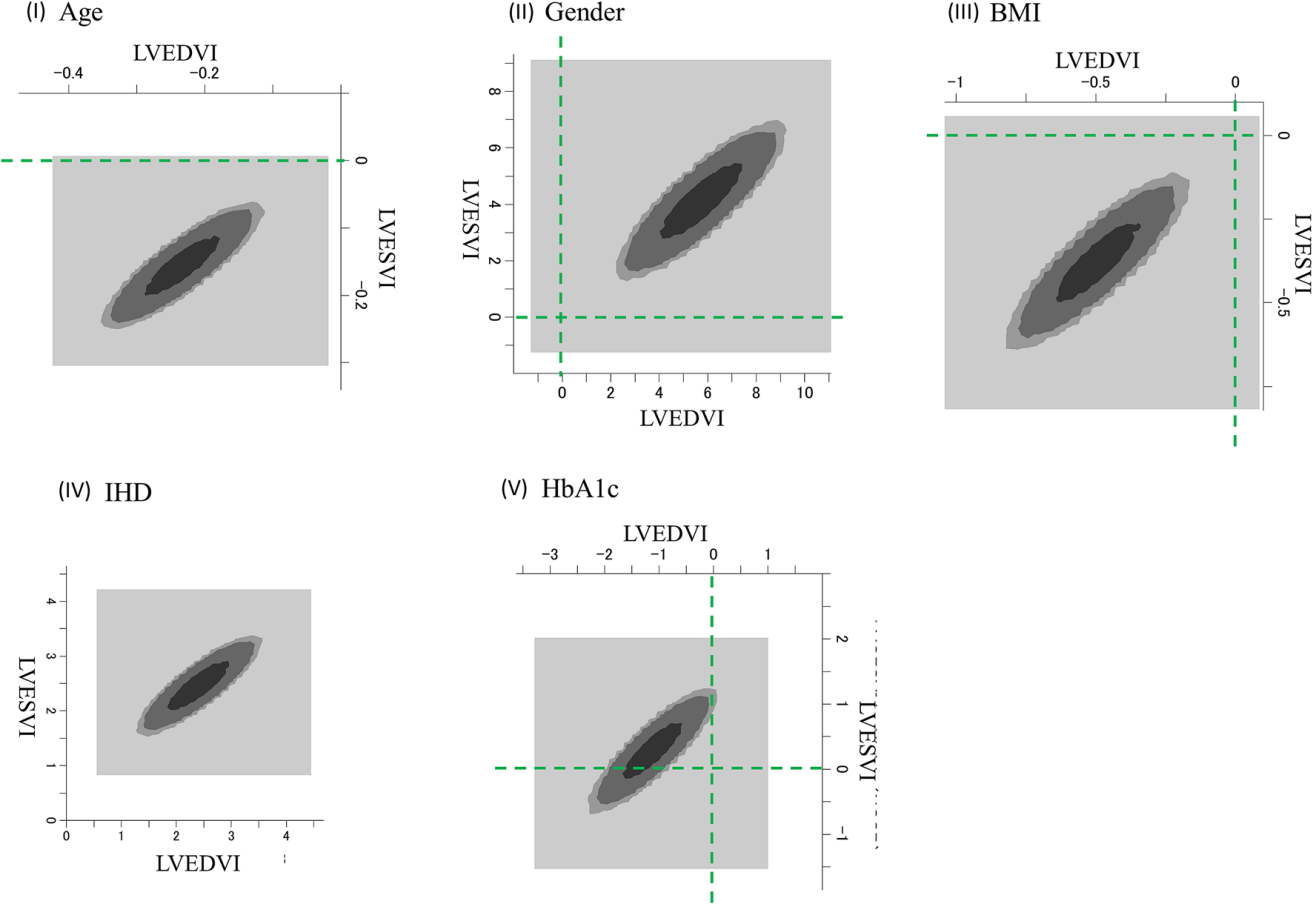
Bayesian SEM documented by 2D contour line. The frequency polygon was described with the marginal posterior distributions of the estimands. The selected 2D contour line was applied in this study. In each figure, the effect of one risk factor on LVESVI is shows on the longitudinal axis. The effect of one risk factor on LVEDVI is shows on the horizontal axis. (I) Age reduced LVESVI and LVEDVI. (II) Male sex increased LVESVI and LVEDVI. (III) BMI reduced LVESVI and LVEDVI. (IV) Vessel disease (severely stenosed coronary arteries) increased LVESVI and LVEDVI. (V) HbA1c decreased LVEDVI but not LVESVI. *SEM* structure equation modeling, *LVESVI* left ventricular end-systolic volume index, *LVEDVI* left ventricular end-diastolic volume index, *BMI* body mass index, *HbA1c* hemoglobin A1c.

Bayesian SEM was applied in this study. A selected two-dimensional (2D) plot of the bivariate posterior density is shown in Fig. 4. Ranging from dark to light, the three shades of gray represent 50%, 90%, and 95% credible regions, respectively. The figures are the results of aging, gender difference, BMI, vessel disease, and HbA1c, which were significantly associated with LVESVI or LVEDVI, as shown in path model A. Note, however, that HbA1c had no effect on LVESVI.

### Additive effects of risk factors

We evaluated the additive effects of the risk factors. We examined four risk factors (female, aging, obesity and diabetes) that itself significantly changed left ventricular cavity size. The risk factors significantly decreased the left ventricular cavity size as the number of risk factor increased (P < 0.001) (show in the Supplementary Figure).

## Discussion

IHD, which is commonly caused by several coronary risk factors, can induce cardiac dysfunction and heart failure. On the other hand, it is poorly understood whether the respective risk factors per se contribute to the progression of cardiac dysfunction or cardiac remodeling. Therefore, this study examined this possibility using SEM. To our knowledge, this study is the first trial to determine the direct influences of respective risk factors on LV cavity sizes in one equation model. Bayesian SEM reassessed the current data from a different statistical perspective. As a result, this study clearly demonstrated that respective risk factors per se contributed to LV cavity sizes in diverse ways. Remarkably, diabetes may be a unique property compared with other risk factors probably because diabetes reduced both systolic and diastolic function.

The precise mechanisms of the diverse relationship between coronary risk factors and LV cavity sizes are still unknown. However, the possible reasons are conceivable as follows.

First, aging is a key factor for modulating LV cavity sizes. Importantly, this study revealed that aging decreases LV cavity sizes at systole and diastole, respectively, although LVEF was rather increased with aging. This indicates that systolic function was preserved and that diastolic function was reduced, because LV enlargement was restricted at diastole. From another perspective, the age-related pathological condition might contribute to heart shrinkage. Age-related interstitial fibrosis in the LV might have made the LV smaller. As for the molecular mechanism, it has been recently reported that poly(A) tail length shortening suppresses *Pabpc1* mRNA translation in mature cardiomyocytes, thereby reducing overall protein synthesis rates in the adult heart^15^.

Second, an increase in BMI suppressed LVESVI and LVEDVI. This may be a simple result of an isolated abnormality of body weight gain. However, the suppression of LV enlargement at diastole would be a serious problem because the “small heart being contrary to the large whole body” may lead to an absolutely low cardiac output, even if systolic function evaluated by LVEF is increased in high BMI as a compensatory mechanism. Adequate blood supply for a lot of oxygen and energy compatible for a large body is required; subsequently, the heart condition is deteriorated early or late^16^. Also, obesity-related factors, such as adipocytokines and other neurohumoral factors, may contribute some harmful effects to the heart^17,18^.

Third, diabetes played a crucial factor in reducing LVEDVI, whereas it did not affect LVESVI. Importantly, LVEF was, of course, reduced in such a situation. Thus, it may be clarified that diabetes has a discriminating characteristic, because diabetes suppresses both systolic and diastolic function. In this respect, diabetes may be a peculiar risk factor for possible future heart failure^18–20^. The causes of remodeling in diabetes cover a lot of ground. Among them, diabetes may be related to cardiac steatosis, impaired myocardial energetics, and enhanced oxidative stress, among others^21^. Regulation of risk factors is naturally important to prevent the onset of IHD; however, this study indicates that risk factor control would yield a direct benefit in the possible prevention of heart failure. We especially believe that control of diabetes is key for prevention of heart failure.

Fourth, it is interesting to see the effect of gender difference on cardiac function and sizes. Simply stated, the size of the heart was bigger with reduced LVEF in males compared with females. It would be safe to say that systolic function tends to be reduced in males than in females. From another perspective, the diastolic function may likely be reduced in females than in males. It is difficult to say which way of viewing the situation should be accentuated between males and females; however, we believe that respective patterns are important characteristically. The current results are in almost agreement with the previous reports^22,23^.

**Figure 5:**
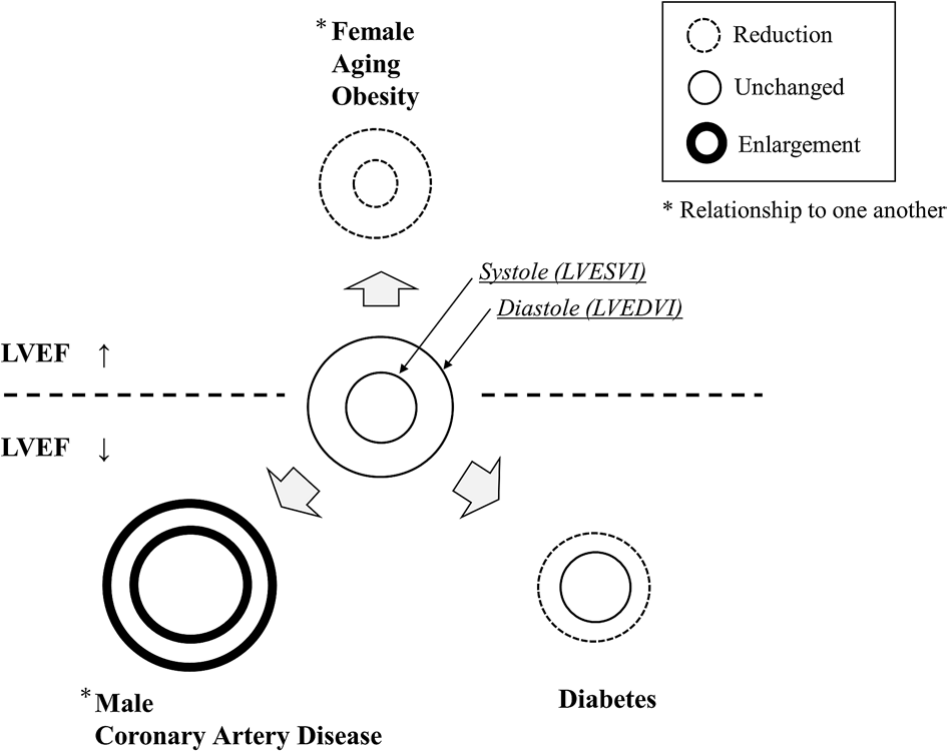
Conceptual scheme. Aging and obesity reduced LV cavity sizes at the systolic and diastolic phases, respectively. Male sex and the severity of coronary artery disease increased the LV cavity size at the systolic and diastolic phases, respectively. Diabetes reduced the LV cavity size only at the diastolic phase but not at the systolic phase, which is a unique property. As for systolic function, female sex, aging, and obesity increased LVEF, and male sex, severity of coronary artery disease, and diabetes reduced LVEF. *LVEF* left ventricular ejection fraction.

Fifth, we investigated whether the accumulation of risk factors alters the morphology of the heart (shown in the Supplementary Figure). As a result, it was suggested that the accumulation of each risk factor may promote changes in the morphology of the heart. The impact of each risk factor may not be strong. However, their combined risks gradually affect the morphology of the heart. In some cases, this can lead to decreased cardiac output and diastolic dysfunction, resulting to the development of heart failure. Finally, a schematic illustration of our findings is shown in Fig. 5.

### Study limitations

First, we did not assess the effect of renal function on LV cavity sizes in this study because renal failure is not necessarily listed as a risk factor of IHD in general. Even so, by an additional analysis, we preliminarily examined a similar analysis that included the serum creatine (Cr) levels in a path model. As a result, although renal dysfunction with high serum Cr level significantly increased LVESVI and LVEDVI, the main data were almost coincident with the present one (precise data not shown). Second, this study was performed mainly among patients with IHD; a similar analysis should be done in other population groups, such as subjects without cardiovascular disease. Third, the medicines that were used in the study patients could not fully be considered. Therefore, we tried to perform further analyses with the addition of medicine (ACE inhibitor and β-blocker) by SEM. The results were similar to those shown in the text (precise data not shown). In addition, the severity of each risk factor, duration of that, and how it was controlled could not be examined by our database. These points will need to be examined in the future. Finally, other risk factors should be discussed in a like manner in the future.

## Conclusions

Female sex, aging, and obesity reduced the systolic and diastolic cavity sizes, with an increase in systolic function. Male sex and a high number of stenosed coronary arteries increased systolic and diastolic cavity sizes, with a decrease in systolic function. However, diabetes reduced diastolic cavity size but did not affect systolic cavity size, with a decrease in systolic function. Thus, this study clearly demonstrated that respective risk factors directly influenced LV cavity sizes in diverse ways. Among them, diabetes directly deteriorated both systolic and diastolic function, which may be a unique property.

## Methods

### Study patients

The study population consisted of 1556 consecutive patients admitted to the Jikei University Hospital from February 2012 to May 2018 in whom left heart catheterization, including hemodynamic measurements, coronary angiography and left ventriculography. We excluded patients who underwent hemodialysis because their cardiac function was significantly altered by artificial volume control. Emergency cases (i.e., acute coronary syndrome) were also excluded, as their hemodynamics (including LVEF) and miscellaneous biomarkers are highly variable and do not reflect the values under stable conditions^10^. Finally, valvular disease was excluded as it is affected by pressure and volume load of the left ventricle. The ethics committee of the Jikei University School of Medicine approved the study protocol (24-355[7121]), and we complied with the routine ethical regulations of our institution. Informed consent was obtained from each patient, and all clinical investigations were conducted in accordance with the principles expressed in the Declaration of Helsinki. According to our routine ethical regulations, we also posted a notice about the study design and contact information at a public location in our institution.

### Disease definitions

IHD was diagnosed based on symptoms, electrocardiography, blood sampling, and coronary artery morphology. Organic lesions producing ≥ 75% luminal stenosis of the coronary arteries on coronary angiography were defined based on the modified American Heart Association coronary tree segment classification. The number of diseased vessels was counted as the number of three major coronary arteries (i.e., left anterior descending, left circumflex, and right coronary arteries) with organic lesions that were indicated for treatment by revascularization and/or standard medical therapy. Diagonal and high lateral branches were included in the left anterior descending and left circumflex arteries, respectively, if they had a substantial myocardial perfusion area and were indicated for treatment. In the present study, we divided the patients into four groups based on the number of diseased vessels with significant organic stenosis (0-, 1-, 2-, and 3-vessel groups). Patients with a left main trunk lesion were included in the 2-vessel group. The number of diseased vessels was counted at the time of cardiac catheterization in this study, and lesions already treated by revascularization were not included. Some of the patients had comorbid cardiovascular diseases, such as valvular disease, arrhythmia, cardiomyopathy, and other conditions. Hypertension, diabetes mellitus, and dyslipidemia were defined as described previously^5–11^.

### Blood sampling and hemodynamic examinations during cardiac catheterization

Blood samples and hemodynamic data were collected during cardiac catheterization. The serum biochemical analyses were performed in a central laboratory of our hospital. We researched smoking habit (0: nonsmoker, 1: past smoker, 2: current smoker).

### Analyses of left ventriculography

Analyses of left ventriculography (QAngioXA V.7.3; Medis, Leiden, The Netherlands) was conducted by expert cardiologists. First, the image sequences were analyzed automatically. In the LV end-diastolic volume (LVEDV) frame, the upper valve point, lower valve point, and apical point had to be selected. After again identifying the upper valve point, lower valve point, and apical point in the LV end-systolic volume (LVESV), the program automatically started the automatic delineation of both LVEDV and LVESV frames. Generated contours could be edited by redrawing unsatisfactory contour regions manually. Consequently, the LVEDVI, LVESVI and LVEF were calculated. The acquired results are of a clinically acceptable quality, and the inter‐ and intra‐observer variabilities are reduced with this automated approach^24^. In addition, we investigated the intra- and inter-observer variability of analyses of left ventriculography in our institution. The standard error (SE) of intra-observer variability was 0.10 for the LVEDVI, 0.08 for the LVESVI and 0.11 for the LVEF. The coefficient of variability (CV) of intra-observer variability was 1.5% for the LVEDVI, 4.6% for the LVESVI and 1.4% for the LVEF. Finally, the SE of inter-observer variability was 0.22 for the LVEDVI, 0.26 for the LVESVI and 0.34 for the LVEF. The CV of inter-observer variability was 0.9% for the LVEDVI, 4.2% for the LVESVI, and 1.3% for the LVEF. The results were a clinically acceptable quality.

### Statistical analyses

Continuous variables are expressed as the mean ± standard deviation or medians. The correlation between two factors was investigated by a single regression analysis and expressed as Spearman’s correlation coefficient. A multiple regression analysis was performed to compare multiple values. The additive effects of risk factors were investigated using a one-way analysis of variance (ANOVA). The above-mentioned statistical analyses were performed using the SPSS Statistics software program (version 25.0, SPSS Inc., Chicago, IL, USA). *P* values of < 0.05 were considered to indicate statistical significance.

A path analysis based on SEM was used to investigate the relationship between clinical factors in this study population and to survey the probable causal effects on the LV cavity sizes or LVEF. The path analysis was performed using the IBM SPSS AMOS software program (version 25, Amos Development Corporation, Meadville, PA, USA). We have previously described how to write a path model^5–14^. In brief, the current models show the relationship between clinical factors and the LV cavity sizes or LVEF using some hierarchical regression models. For every regression, the total variance in dependent variable is theorized to be affected by either independent variables that are included in the model or by extraneous variables (e). The structural equation models that were obtained were tested and confirmed; *P* values of < 0.05 were considered to indicate statistical significance.

In addition, we applied Bayesian SEM by using a program embedded in IBM SPSS AMOS (version 25.0). This approach replaces parameter specifications of exact zeros and exact equalities with approximate zeros and equalities based on informative, small variance priors. This will produce analytical results that better reflect substantive theory. We believe that additional testing by Bayesian SEM would be rationalized and helpful to reassess our data from a different angle of statistics. In IBM SPSS AMOS, the summary table in the Bayesian SEM window becomes available. The frequency polygon was described with the marginal posterior distributions of the estimands. A 2D contour line was applied in this study because it is easily visualized.

## Supplementary Information

Supplementary Figure.

## References

[1] Delicce, A. V. & Makaryus, A. N. In *StatPearls* (StatPearls Publishing LLC, 2018).

[2] Khera AV (2016). Genetic risk, adherence to a healthy lifestyle, and coronary disease. N. Engl. J. Med..

[3] Takaoka K (2000). Comparison of the risk factors for coronary artery spasm with those for organic stenosis in a Japanese population: Role of cigarette smoking. Int. J. Cardiol..

[4] Petersen SE (2017). The impact of cardiovascular risk factors on cardiac structure and function: Insights from the UK Biobank imaging enhancement study. PLoS ONE.

[5] Kinoshita K (2016). Potent influence of obesity on suppression of plasma B-type natriuretic peptide levels in patients with acute heart failure: An approach using covariance structure analysis. Int. J. Cardiol..

[6] Tsutsumi J (2017). Manifold implications of obesity in ischemic heart disease among Japanese patients according to covariance structure analysis: Low reactivity of B-type natriuretic peptide as an intervening risk factor. PLoS ONE.

[7] Ogawa K (2017). Parallel comparison of risk factors between progression of organic stenosis in the coronary arteries and onset of acute coronary syndrome by covariance structure analysis. PLoS ONE.

[8] Ito S (2017). Possible increase in insulin resistance and concealed glucose-coupled potassium-lowering mechanisms during acute coronary syndrome documented by covariance structure analysis. PLoS ONE.

[9] Yoshida J (2017). Associations between left ventricular cavity size and cardiac function and overload determined by natriuretic peptide levels and a covariance structure analysis. Sci. Rep..

[10] Tanaka Y (2017). Close linkage between serum uric acid and cardiac dysfunction in patients with ischemic heart disease according to covariance structure analysis. Sci. Rep..

[11] Fukumoto R (2017). Conflicting relationship between age-dependent disorders, valvular heart disease and coronary artery disease by covariance structure analysis: Possible contribution of natriuretic peptide. PLoS ONE.

[12] Sugawa S, Masuda I, Kato K, Yoshimura M (2018). Increased levels of cardiac troponin I in subjects with extremely low B-type natriuretic peptide levels. Sci. Rep..

[13] Oki Y (2019). High serum uric acid is highly associated with a reduced left ventricular ejection fraction rather than increased plasma B-type natriuretic peptide in patients with cardiovascular diseases. Sci. Rep..

[14] Itakura R (2020). A highly-sensitized response of B-type natriuretic peptide to cardiac ischaemia quantified by intracoronary pressure measurements. Sci. Rep..

[15] Chorghade S (2017). Poly(A) tail length regulates PABPC1 expression to tune translation in the heart. eLife.

[16] Khan SS (2018). Association of body mass index with lifetime risk of cardiovascular disease and compression of morbidity. JAMA Cardiol..

[17] Kaneda H (2018). Association of serum concentrations of irisin and the adipokines adiponectin and leptin with epicardial fat in cardiovascular surgery patients. PLoS ONE.

[18] von Jeinsen B (2018). Association of circulating adipokines with echocardiographic measures of cardiac structure and function in a community-based cohort. J. Am. Heart Assoc..

[19] Pavlovic A (2018). Long-term mortality is increased in patients with undetected prediabetes and type-2 diabetes hospitalized for worsening heart failure and reduced ejection fraction. Eur. J. Prev. Cardiol..

[20] Aune D (2018). Diabetes mellitus, blood glucose and the risk of heart failure: A systematic review and meta-analysis of prospective studies. Nutr. Metab. Cardiovasc. Dis. NMCD.

[21] Nagoshi T, Yoshimura M, Rosano GM, Lopaschuk GD, Mochizuki S (2011). Optimization of cardiac metabolism in heart failure. Curr. Pharm. Des..

[22] Kishi S (2015). Race-ethnic and sex differences in left ventricular structure and function: The Coronary Artery Risk Development in Young Adults (CARDIA) Study. J. Am. Heart Assoc..

[23] Mohammed SF (2012). Comorbidity and ventricular and vascular structure and function in heart failure with preserved ejection fraction: A community-based study. Circ. Heart Fail..

[24] Oost E, Oemrawsingh P, Reiber JH, Lelieveldt B (2009). Automated left ventricular delineation in X-ray angiograms: A validation study. Catheter. Cardiovasc. Interv..

