# Supplementary material for: Possible diverse contribution of coronary risk factors to left ventricular systolic and diastolic cavity sizes
Source: Sci Rep. 2021 Jan 15;11:1570. doi: 10.1038/s41598-021-81341-1 (PMC7810980; doi:10.1038/s41598-021-81341-1)
Supplement: Supplementary file 1 — Supplementary Figure. [file 41598_2021_81341_MOESM1_ESM.docx]

**Supplementary Information**

**Possible Diverse Contribution of Coronary Risk Factors to Left Ventricular Systolic and Diastolic Cavity Sizes**

Kenichiro, Suzuki, MD*, Yasunori Inoue*, MD, PhD, Kazuo Ogawa, MD, PhD, Tomohisa Nagoshi, MD, PhD, Kosuke Minai, MD, PhD, Takayuki Ogawa, MD, PhD, Makoto Kawai, MD, PhD, Michihiro Yoshimura, MD, PhD

*Both authors contributed equally to this manuscript.

Division of Cardiology, Department of Internal Medicine, The Jikei University School of Medicine, 3-25-8 Nishi-shinbashi, Minato-ku, Tokyo 105-8461, Japan.

**Address for correspondence**

Corresponding author: Yasunori Inoue, MD, PhD

Division of Cardiology, Department of Internal Medicine, The Jikei University School of Medicine, 3-25-8 Nishi-shinbashi, Minato-ku, Tokyo l05-8461, Japan.

Phone: +81-3-3433-1111

Fax: +81-3-3459-6043

E-mail: [y.inoue@jikei.ac.jp](about:blank)

**Authors’ email addresses**

Kenichiro Suzuki, MD: ken-suzuki@jikei.ac.jp

Kazuo Ogawa, MD, PhD [oga-n@jikei.ac.jp](about:blank)

Tomohisa Nagoshi, MD, PhD [tnagoshi@jikei.ac.jp](about:blank)

Kosuke Minai, MD, PhD [heart@jikei.ac.jp](about:blank)

Takayuki Ogawa, MD, PhD [takaog39@jikei.ac.jp](about:blank)

Makoto Kawai, MD, PhD [cadmk@jikei.ac.jp](mailto:cadmk@jikei.ac.jp)

Michihiro Yoshimura, MD, PhD [m.yoshimura@jikei.ac.jp](about:blank)


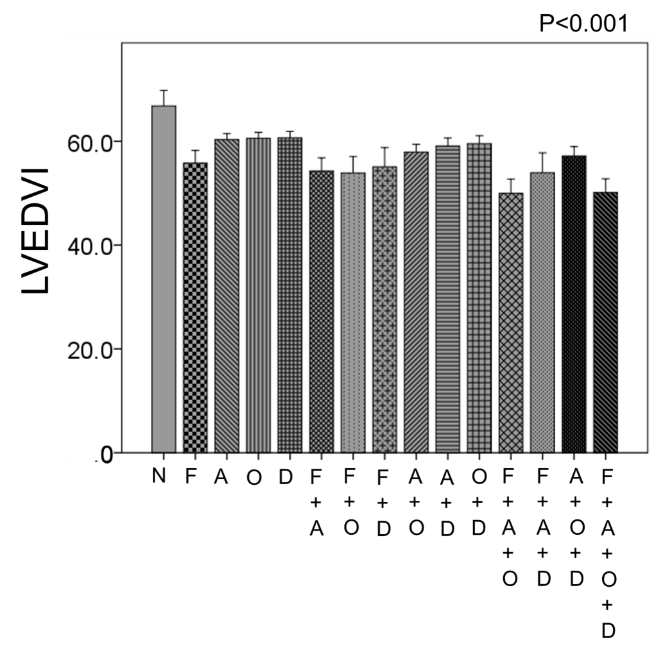

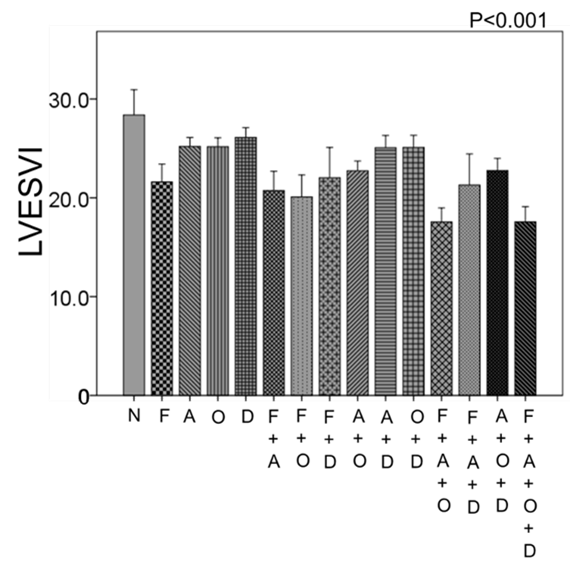


**Supplementary Figure. The additive effects of risk factors (female, aging, obesity and diabetes).**

The additive effects of risk factors (female, aging, obesity and diabetes) are shown. These risk factors significantly decreased the left ventricular cavity size as the number of risk factors increased.

LVEDP, left ventricular end-diastolic pressure; LVEF, left ventricular ejection fraction; LVEDVI, left ventricular end-diastolic volume index, N, no risk; F, Female; A, Aging; O, Obesity; D, Diabetes.
